# Facial Expressions of Horses Using Weighted Multivariate Statistics for Assessment of Subtle Local Pain Induced by Polylactide-Based Polymers Implanted Subcutaneously

**DOI:** 10.3390/ani12182400

**Published:** 2022-09-13

**Authors:** Júlia R. G. Carvalho, Pedro H. E. Trindade, Gabriel Conde, Marina L. Antonioli, Michelli I. G. Funnicelli, Paula P. Dias, Paulo A. Canola, Marcelo A. Chinelatto, Guilherme C. Ferraz

**Affiliations:** 1 Department of Animal Morphology and Physiology, School of Agricultural and Veterinarian Sciences, São Paulo State University, FCAV/UNESP, Jaboticabal 14884-900, SP, Brazil; 2 Department of Veterinary Surgery and Animal Reproduction, School of Veterinary Medicine and Animal Science, São Paulo State University, FMVZ/UNESP, Botucatu 18618-681, SP, Brazil; 3 Department of Veterinary Clinical and Surgery, School of Agricultural and Veterinary Sciences, São Paulo State University, FCAV/UNESP, Jaboticabal 14884-900, SP, Brazil; 4 Department of Technology, School of Agricultural and Veterinary Sciences, São Paulo State University, FCAV/UNESP, Jaboticabal 14884-900, SP, Brazil; 5 Department of Materials Engineering, São Carlos School of Engineering, University of São Paulo, EESC/USP, São Carlos 13563-120, SP, Brazil

**Keywords:** animal welfare, behavior, equine, validation, principal component analysis

## Abstract

**Simple Summary:**

Facial expression (FE) has been used for pain diagnosis in horses. The current study aimed to identify pain in horses undergoing under-skin polylactide-based polymer implantation. Five statistical methods for analyzing FE were used, including conventional and new approaches. First, we scored the seven FEs separately. Subsequently, the scores of the seven FEs were added (SUM). Subsequently, principal component analysis (PCoA) was performed using the scores of the seven FEs obtained using the first method. Afterwards, weights were created for each FE based on each variable’s contribution variability obtained from the PCoA (SUM.W). Finally, we applied a general score to the animal’s face (GFS). The horses were filmed before and 24 and 48 h after implantation. The tissue sensitivity to mechanical stimulation and skin temperature of the horses were assessed at the same time points. The results show no changes in the FEs analyzed separately or jointly. The horses with incision and suture but no polymer implant displayed a higher pain-related FE 48 h after implantation, while the horses implanted with polymers displayed more apparent alterations in the mechanical skin sensitivity and temperature. Our findings show that the five statistical methods used to analyze the faces of the horses were not able to detect low-grade inflammatory pain.

**Abstract:**

Facial-expression-based analysis has been widely applied as a pain coding system in horses. Herein, we aimed to identify pain in horses undergoing subcutaneously polylactide-based polymer implantation. The sham group was submitted only to surgical incision. The horses were filmed before and 24 and 48 h after implantation. Five statistical methods for evaluating their facial expressions (FEs) were tested. Primarily, three levels of scores (0, 1, and 2) were applied to the seven FEs (ear movements, eyebrow tension, orbicularis tension, dilated nostrils, eye opening, muzzle tension, and masticatory muscles tension). Subsequently, the scores of the seven FEs were added (SUM). Afterwards, principal component analysis (PCoA) was performed using the scores of the seven FEs obtained using the first method. Subsequently, weights were created for each FE, based on each variable’s contribution variability obtained from the PCoA (SUM.W). Lastly, we applied a general score (GFS) to the animal’s face (0 = without pain; 1 = moderate pain; 2 = severe pain). The mechanical nociceptive threshold (MNT) and cutaneous temperature (CT) values were collected at the same moments. The results show no intra- or intergroup differences, when evaluating each FE separately or in the GFS. In the intragroup comparison and 48 h after implantation, the control group showed higher values for SUM, PCoA, and SUM.W, although the horses implanted with polymers displayed more obvious alterations in the CT and MNT. Our findings show that the five statistical strategies used to analyze the faces of the horses were not able to detect low-grade inflammatory pain.

## 1. Introduction

In recent decades, global society has shown more significant concern and care for the quality of life of non-human animals, in line with significant advances in the science of behavior and animal welfare [1]. The diagnosis or accurate quantification of a painful sensation represents a semiotic challenge due to the multidimensionality of pain, constituted by the combination of somatic, cognitive, and emotional components [2,3,4].

Better and early pain recognition and control are essential to improve welfare [5], as adequate pain management depends on the accuracy of pain assessment [6]. For pain diagnosis, physiological indicators such as heart rate, respiratory rate, and blood cortisol concentration could be used. However, these parameters are not specific to pain and can be changed due to many other situations. Thus, non-verbal communication, such as body language, has higher specificity and sensitivity to detect pain [7]. The behavioral expression of pain replaces the lack of verbal expression in animals [8] and is a useful tool because it is a non-invasive, non-intrusive, and practical method [9,10].

Studies have investigated pain based on behavior, showing generalizable indicators for several species (e.g., appetite, locomotion, activity). The behavior can also be species-specific, such as horses pawing, pigs wagging their tails intensely and continuously, and cattle lying in a prone position with the full or partial extension of one or both hind limbs [11,12,13]. A study recently showed that the spontaneous blink rate could also be a reliable method to measure stress and attention in horses subjected to a controlled “sham clipping” (sight and sound of hair clippers) [14]. Although many studies focus on identifying pain and other types of stressful stimuli in animals, no method is considered the gold standard for horses [15,16].

The challenge of measuring the degree of pain in animals is similar to that of measuring pain in human patients who have difficulty with verbal communication. One solution to this restriction was the creation of an ethogram of human facial tensions and contractions (Facial Action Coding System; FACS; [17]), which has presented promising results in detecting pain in individuals with dementia [18] and in children [19]. In recent years, studies with non-human animals have followed this medical trend. Therefore, enormous effort has been invested in recognizing subtle behavioral changes associated with pain in animals. Using a new approach, facial expressions have been identified in several species, and scales have been developed to measure pain in mice [20,21,22,23], rats [24,25,26], rabbits [27,28,29,30], ferrets [31], seal [32], cats [33,34], pigs [35,36,37,38], cattle [39,40,41], and sheep [8,42,43,44].

Facial features in horses have been studied after surgical castration procedures [45], in animals with acute colic syndrome [46], in pain inducement with the use of a tourniquet on the foreleg and a topical application of capsaicin [47], in animals with acute laminitis [48], in horses mounted during competitions [5,49], in the induction of positive emotions [50], and in the evaluation of the effect of exercise on ranch horses [51]. Recently, studies have used the face to evaluate dental disorders [52,53], the calming effect of aromatherapy [54], the effect of transportation and social isolation [55], sedation [56], and induced orthopedic pain [4,57]. Regarding pain, in general, studies have identified facial changes in horses subjected to moderate to severe pain conditions [45]. However, studies examining horses’ faces in clinical situations of subtle or mild pain are incipient.

Of all the studies that have examined facial changes in horses in painful situations, only one applied traditional methods to identify inflammation and local sensitivity [47]. The mechanical nociceptive threshold test may be performed using von Frey filaments, which reveals a nociceptive threshold used to mimic clinical conditions that present increased skin sensitivity, such as neuropathic pain, postoperative pain, inflammation, or osteoarthritis [58,59]. Furthermore, tissue lesions induce vasodilation and an increase in cellular exothermic metabolism, causing an increase in temperature at the injury site, a classic inflammation sign. The emission of heat from the body surface can be captured by infrared thermography, which may provide quantitative data on the degree of inflammation of the underlying tissues [60].

Despite the capacity of facial scales to discriminate between “pain-free” and “painful”, the developers of one scale questioned the ability to quantify pain using these instruments [47]. Facial expressions can present complex interpretations. For example, dilated nostrils are interpreted as postoperative pain [45] and orthopedic pain [57]. However, they were seen during transportation and social isolation [55] as well as after physical exercise, due to hyperventilation [51]. Another more complex example is partially or entirely closed eyes, which occurred after castration, being interpreted as pain [45], but which were also observed after physical exercise as signs of physical tiredness [51] and relaxation [50]. Closed eyes interpreted as pain can be related to exhaustion, due to the anesthetic and surgical procedure, and not exclusively to the pain process [47]. This characteristic is also expected to be displayed when the horse is asleep or resting [61]. Thus, it is evident that the same specific behavior can be exhibited due to different sensations.

This evidence suggests that a facial feature analyzed in isolation may result in a false-positive diagnosis or an overestimation of pain intensity. The recent literature reviews have indicated facial configuration analyses, represented by the joint assessment of facial features using multivariate statistical techniques [62,63], which have not yet been explored by previous studies. In this sense, it is possible that some facial features are more relevant than others in specific scenarios (different painful experiences, physical activity, rest, postoperative, etc.) [64], and that weights can be assigned to facial features to improve the diagnostic capacity of pain. These considerations have not yet been studied in the evaluation of facial expressions in horses.

Given the aforementioned information, studies have provided robust evidence that horses’ facial evaluations can be used as an important pain coding system [4,5,6,7,45,46,47,48,49,51,53,57,64]. However, it is still uncertain whether facial expressions are sensitive to classify pain quantitatively or if they can identify alterations associated with subtle pain, and if statistical approaches with weightings and multivariate methods can contribute to the quantification of pain.

In the present study, we investigated whether facial expressions could identify pain in horses submitted to subcutaneous implantation of polylactide-based materials, using different statistical strategies to analyze the face during the pain evaluation. We hypothesized that the evaluation of facial expressions allows for the diagnosis of facial expressions of horses, using weighted multivariate statistics to assess low-grade inflammatory pain in horses undergoing subcutaneous implantation of polylactide-based polymers, and that weighted multivariate approaches can diagnose subtle pain. It is important to highlight that this study is an opportunistic study, and the polymers used herein were previously tested to assess their biocompatibility and biodegradation in horses and were safe, with potential for use in equine medicine [65].

## 2. Materials and Methods

### 2.1. Ethics in the Use of Animals

All procedures performed followed the Ethical Principles in Animal Experimentation adopted by the National Council for Control in Animal Experimentation (CONCEA). The protocol was reviewed and approved by the Ethics Committee on the Use of Animals (CEUA, UNESP, Jaboticabal, Brazil (Protocol no. 006548/17)).

### 2.2. Preparation of Polymers

The blends were prepared according to Dias and Chinelatto (2019) [66]. A mixing process was carried out in the molten state, containing PLA Ingeo 3251D (Nature Works, Plymouth, MN, USA), PCL CapaTM 6500 (Perstorp, Malmö, Sweden), and compatibilizer Capa 7201ª (Perstorp, Malmö, Sweden). Before processing, the PLA was dried at 80 °C for 8 h in an air-circulating oven, and at 40 °C in a vacuum oven for at least 16 h. After drying the polymers, manual mechanical pre-mixing of the dried PLA/PCL granules with the compatibilizing agent was performed. The implants were made using hot pressing at 180 °C, with dimensions of 1 cm square (cm^2^) and 1 mm thick, and were sterilized with ethylene oxide [65].

### 2.3. Animals

Six adult mixed breed horses were used, three male and three female, with an average weight of 405 ± 37 kg and aged between 10 and 18 years, from the didactic herd of the Laboratory of Equine Exercise Physiology and Pharmacology (LAFEQ) at the Department of Animal Morphology and Physiology, FCAV/UNESP—Jaboticabal campus. The animals were kept in a paddock and fed 0.2% of their body weight in mash ration once a day, in addition to silage, mineral salt, and water ad libitum. Before the beginning of the experimental stages, to determine their health status, the animals were submitted to a complete physical examination. Hematological and biochemical exams were performed and may be observed in our previous study [65]. The horses were previously treated with anthelmintics, which was repeated every four months, and vaccinated against rabies, tetanus, Eastern and Western equine encephalomyelitis, and equine influenza types A1 and A2.

### 2.4. Experimental Groups

As the present work was an opportunistic study, we extracted information from content previously published by our research group [65]. The polymers were implanted subcutaneously on the lateral face of the neck in the prescapular region of the six horses in a balanced, randomized design, with a 14-day washout between implantations. This way, 12 lateral sides of the neck were used for implantation. The first group (PLA; *n* = 6) received a pure PLA implant on the right side of the neck. The second group (PLA/PCL; *n* = 6) received an implantation of the PLA/PCL blend on the left side of the neck. The third group, denoted the sham group (S; *n* = 12), was submitted only to the surgical incision, which was performed bilaterally, similarly to the groups implanted with the mentioned polymers (PLA or PLA/PCL).

In the present study, we used the implantation of the polymers (PLA and PLA/PCL) to form a single group, considering that, in the previous study [65], no changes in the cutaneous temperature or mechanical nociceptive threshold were detected throughout the experimental period between polymers. Thus, we studied two groups: the implanted group (IG; *n* = 12, with the two types of polymers—PLA and PLA/PCL) and the sham group (S; *n* = 12, only incision). The combination of the PLA and PLA/ PCL groups was possible because there was no statistical difference between them (as described below in the statistical analysis section).

The procedures for implanting the biomaterials (PLA or PLA/PCL), as well as obtention of the S group, were performed randomly, and, for the first procedure, two horses were used for the PLA group, two horses for the PLA/PCL group, and two horses for the S group. These procedures were repeated every 14 days until four implantations had been performed in each animal (PLA, PLA/PCL, and S in each lateral surface of the neck). This interval was stipulated so that there was no possible overlap of systemic inflammatory responses from previous implantations, and the plasma fibrinogen biomarker, an acute phase indicator of inflammation, was used as a guide. Thus, the procedures were performed if the fibrinogen concentrations were in the normal reference range for equine species [65].

### 2.5. Procedure for Implantation of Biopolymers

The hair was previously shaved in an area of approximately 8 cm^2^ of the lateral face of the neck, in the prescapular region (right or left). Subsequently, the animals were sedated by intravenous administration of detomidine hydrochloride (0.01 mg∙kg^−1^). An infiltrative anesthetic block around the incision site was performed with 2.0 mL of 2% lidocaine hydrochloride. A 2 cm horizontal skin incision was made with a No. 15 scalpel blade in the determined area on the lateral surface of the neck. Space was obtained between the skin and cutaneous muscle by blunt dissection, where the polymer was implanted. Subsequently, dermorraphy was performed in a simple interrupted pattern with nylon 0. At the end of the implantation procedure, sedation was reversed with the intravenous administration of yohimbine (0.12 mg∙kg^−1^). The postoperative period consisted of cleaning the area using gauze, 0.9% saline, and fly repellent ointment around the surgical wound once a day for 14 days. No type of analgesic and/or anti-inflammatory medication was provided during the experimental period. The stitches were removed on the 7th day after the surgical procedure. All surgical procedures were performed by the same surgeon (MLA).

### 2.6. Facial Expressions Evaluation

The horses’ faces were filmed with a video camera (XA10 A KIT, Canon, SP, Brazil) for 5 min. The horses were placed in a location that they were already acclimatized to and were not contained in horse stocks or tied up. The camera was positioned on a tripod approximately 2 m away from the animal and operated by a researcher familiar with the horses (JRGC). The researcher positioned themselves approximately three meters from the horses without looking at them (directly in the eyes), behaving in the most disinterested way possible. The videos were always recorded in the same place, 1 h before and 24 and 48 h after the implantation procedure. Two 30 s video clips from each evaluation moment were extracted from the raw videos (5 min), which were selected with the condition that there was adequate lighting, a good profile, and minimal external disturbances, thus providing a clear and accurate view of the horses’ heads and facial features to be studied [48]. It is worth mentioning that the editing and randomization of the 30 s video clips were carried out by one of the authors (J.R.G.C.), who did not evaluate the behavior. The two video clips from the same moment were understood as a repetition.

The 30 s video clips were analyzed using ELAN software (ELAN Linguistic Annotator, version 4.9.4, Nijmegen, The Netherlands) by a double-blinded evaluator (PHET), using the continuous focal animal method [67]. The evaluator had experience in horses’ facial evaluation in a previously published study [51]. Before starting the evaluation of the video clips, 10 video clips belonging to the data set were selected randomly (JRGC) and evaluated twice (PHET), with an interval of 30 days, to calculate the intraobserver reliability (repeatability), using the two methodologies described below.

The 30 s video clips were scored using two methodologies. In the first, seven facial features were scored individually, as shown in Table 1. For the individual evaluation of facial features, the evaluator was allowed to watch the video clip as many times as necessary. In the second method, the general expression of the face was measured through an additional evaluation of the video clips, which occurred 30 days after the first method, applying only a general score to the animal’s face with three levels (0 = without pain; 1 = moderate pain; 2 = severe pain), without considering each characteristic separately. It should be mentioned that, for this, the observer analyzed each 30 s video clip only once.

The facial features evaluated in this study were defined based on two previously described pain assessment scales in horses. The first one, described by Gleerup et al. (2015) [48], consists of changes in the position of the ears, eyes, nostrils, and muscle tension. The second scale used was described by Dalla Costa et al. (2014) [46] and comprised facial action units, namely stiffly backwards ears, orbital tightening, tension above the eye area, prominent strained chewing muscles, mouth strained and pronounced chin, strained nostrils, and flattening of the profile.

**Table 1:** Descriptions of the 7 facial features and their scores adapted from Dalla Costa et al. (2014) [45] and Gleerup et al. (2015) [47].

Facial Features	Scores	Descriptions
Ear movements	0	Ears directed forward or to the side in most of the video clip.
	1	Ears directed backwards at least once or in less than half of the video clip.
	2	Ears directed backward in most of the video clip or pressed against the neck at least once.
Eyebrow tension	0	Eyebrows not contracted during the video clip.
	1	Moderately * contracted eyebrows in most of the video clip.
	2	Intensely * contracted eyebrows in most of the video clip.
Orbicularis tension	0	Orbicularis muscle not contracted when blinking during the video clip.
	1	Orbicularis muscle moderately contracted when blinking at least once during the video clip.
	2	Orbicularis muscle intensely ^#^ contracted when blinking at least once during the video clip.
Dilated nostrils	0	Nostrils not dilated in most of the video clip.
	1	Nostrils moderately dilated in most of the video clip.
	2	Nostrils intensely dilated in most of the video clip.
Eye opening	0	Eyes completely open in most of the video clip.
	1	Eyes partially closed in most of the video clip.
	2	Eyes completely closed in most of the video clip.
Muzzle tension	0	Muzzle without tension during the video clip.
	1	Muzzle moderately tense in most of the video clip.
	2	Muzzle intensely tense in most of the video clip.
Masticatory muscles tension	0	Masticatory muscles without tension during the video clip.
	1	Masticatory muscles moderately tense in most of the video clip.
	2	Masticatory muscles intensely tense in most of the video clip.

* The words moderately and intensely are subjective terms that refer to the intensity of a contraction or tension scored according to the evaluator’s expertise based on the duration and number of evident wrinkles (folds). ^#^—Blinking behavior was not included in the characteristic “Orbicularis tension”.

### 2.7. Thermographic Evaluation

The animals were taken to a closed, ventilated area, free from drafts and direct exposure to the sun. The implantation site was cleaned with dry gauze 30 min before the images were taken, and the area was not touched after cleaning. These procedures were performed to allow the animal to acclimatize to the room temperature. The temperature and humidity of the environment were controlled to standardize the thermal measurements. The animals were not sedated during the evaluations. An infrared thermographic camera (model i50, Flir Systems, Wilsonville, OR, United States) was used, with a sensitivity value of less than 0.1 °C, emissivity of 0.98, temperature variation from −20 to 350 °C, and a resolution of 140 × 140 pixels. To obtain thermographic images, the camera was positioned 0.5 m away, perpendicularly to the implantation site. The evaluations were carried out after filming for the facial expression evaluations at the same moments: 1 h before and 24 and 48 h after the procedure. All images were obtained by the same evaluator (JRGC). FLIR Tools software (FLIR Systems Inc, Wilsonville, OR, USA) was used to analyze the images. The images were evaluated by a blinded observer. An area of 10 cm^2^ was drawn on the thermographic image (IG and S), and the evaluations of the thermographic images were performed blindly. The software calculated the mean cutaneous temperature (CT) of the defined area for each moment.

### 2.8. Evaluation of Mechanical Nociceptive Threshold

The mechanical nociceptive threshold (MNT) testing technique has a valuable capacity to recognize altered nociceptive responses. Herein, we used von Frey filaments (FVFs) (Touch-TestTM Sensory Evaluators, Stoelting Company, Wood Dale, IL, USA) to evaluate the cutaneous MNT. In accordance with the guidelines of the manufacturer, six filaments with sizes from 5.07 to 6.65 were utilized, representing an applied force of 11.8 to 446.7 g, respectively. The filaments were applied perpendicularly to the animal’s skin until the nylon thread started to bend. Four applications were performed around the implantation sites at approximately 1 cm, with intervals of 3 s. Initially, the thinnest filament was used, the next filament was used when it was not possible to observe an aversive response, and so on, until the animals demonstrated an aversive response or the largest filament was used. The aversive response was defined as moving the tail, ears, or head, kicking, or stepping to the side (Table 2). Simple reflexes of movement at the first touch of the filament on the skin were not accepted as an aversion response, and, in these cases, the test was repeated after 10 s. The evaluations were carried out immediately after thermographic evaluation, 1 h before and 24 and 48 h after the procedure. The same operator performed all measurements (JRGC), with the horses in a quadrupedal position, in an area without movement restrictions. The values obtained were converted into force (g), in accordance with the table provided by the manufacturer.

### 2.9. Statistical Analysis

All statistical analyses were performed in R software with the RStudio integrated development environment (Version 4.0.2 (2020-06-22), RStudio, Inc., Auckland, New Zealand) (PHET), presenting the functions and packages used in the format “function {package}”. For all analyses, an α of 5% was considered. A priori, three analyses were performed as prerequisites for analyzing the database. The experimental groups from a previous study [65] performed by our team (PLA, PLA/PCL, and S) were compared at each time of assessment by the Kruskal–Wallis test (kruskal {agricolae}). The repeatability of the evaluator’s score was verified with the intraobserver reliability by applying the quadratic weighted kappa coefficient (cohen.kappa {psych}) and the test retest with the two-tailed paired Wilcoxon test (stats {wilcox.test}) among the 30 s video clips extracted from the same video and moment (repetition).

**Figure 1:**
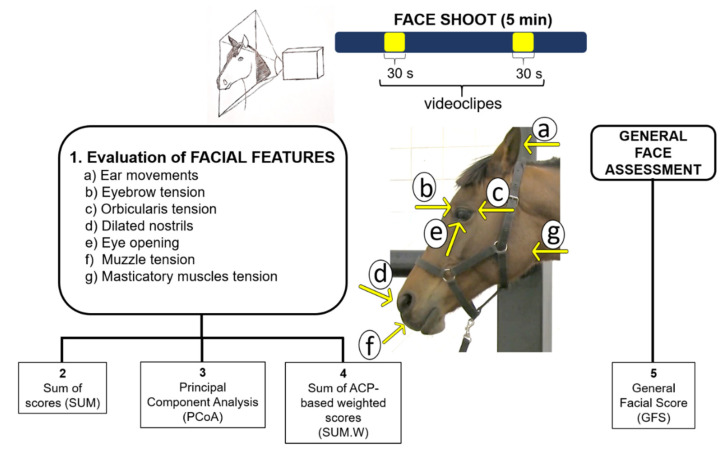
Infographic presenting the strategies used to evaluate facial expressions in horses submitted to polymer implantation.

Subsequently, the facial expressions were evaluated using five different strategies (Figure 1). In the first strategy, the seven facial features were assessed individually. In the second (SUM), the scores of the seven facial features were added together. In the third strategy, principal component analysis (PCoA; princomp {stats}) was performed using the scores of the seven facial features of all groups and moments together, and the scores of the PCoAs for each animal (get_pca_ind {factoextra}) from the dimension representative were extracted (eigenvalue > 1 and variance > 20%; in our case, only the first dimension). The score of the PCoA algorithm is a vector that concentrates the information of all the variables included in the analysis, referring to the same dimension in a single value that can be understood as an index [68]. The load values were also calculated for each variable in the first two dimensions (get_pca_var {factoextra}). In the fourth strategy (SUM.W), weights were estimated for each facial feature based on the load value of each variable obtained from the PCoA in the first dimension. Thus, the load value of the variables in the first dimension was subtracted from the square root of the corresponding eigenvalue, thus extracting a weight for each variable [69]. Next, the gross scores of the seven features were added to the estimated weights, and a new sum was performed. Finally, the fifth strategy (GFS) consisted of awarding a general score for the faces of the horses without considering individual facial features.

Intragroup and intergroup comparisons over time were performed by analysis of variance applying mixed linear models (lmer {lme4}) for the models that presented the Gaussian residual distribution (resid {stats}) and were analyzed by histograms (hist {stats}), box graphics (boxplot {graphics}), and quantile–quantile (qqnorm {stats}). When the assumption of residual normality was not met, generalized mixed linear models were applied (glmer {lme4}). In both cases, the moments and the side of the neck were included in the models as fixed effects, and the random effects included the individual and the repetition, using Tukey’s test as a post hoc analysis (lsmeans {lsmeans}). Finally, to evaluate the relationship between the strategies adopted to evaluate the facial expressions, the MNT, and the CT, Spearman’s rank correlation coefficient was used (rcorr {Hmisc}), considering all the moments and experimental groups together. Exclusively for the correlation, the MNT and CT information was duplicated to match the number of behavioral measurements, which were collected twice for each animal at the same time (repetition for the test retest).

The heatmap method is a practical method for evaluating the grouping and determining the relationships among the variables. Heatmap and hierarchical clustering were performed using the ‘pheatmap’ function with default parameters (clustering_distance_cols = euclidean, clustering_method = complete) implemented in R (v.3.6.3) (R Core Team, Vienna, Austria).

## 3. Results

The experimental groups (PLA, PLA/PCL, and S) from our previous study presented no statistical differences between the current study’s moments. The repeatability test was performed to evaluate whether the observer presented consistency in the assessments. The intraobserver reliability was classified as good to very good. Very high agreements (k = 0.80 to 1.0) were found for 71% of the variables, while substantial agreements (k = 0.60 to 0.79) were found for 29% of the variables (Table 3). There was no difference between the scores of the two video clips of the same animal at the same moment (test retest) (Table 4).

**Figure 2:**
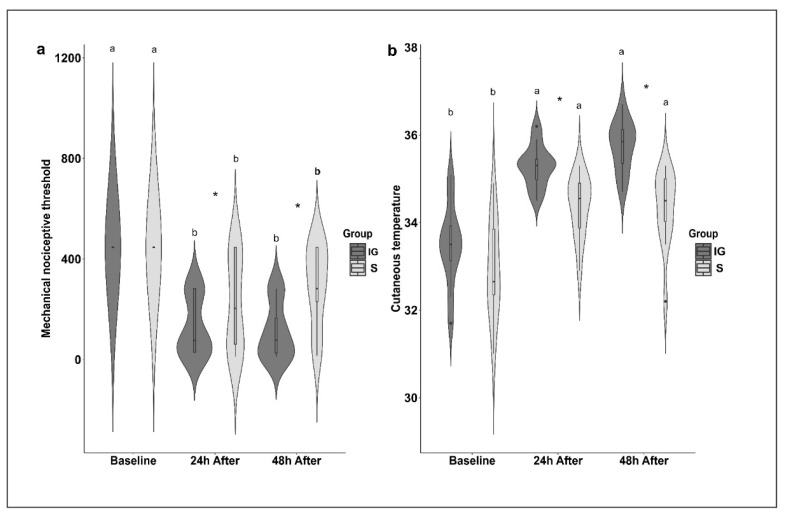
Violin plot of medians and amplitude of the (**a**) mechanical nociceptive threshold (MNT) and (**b**) the cutaneous temperature (CT) of horses submitted to polymer implantation. IG, implanted group; S, sham group—skin incision only. Different letters indicate differences over time for the same group. * Indicates difference between groups at the same time (*p* < 0.05).

**Figure 3:**
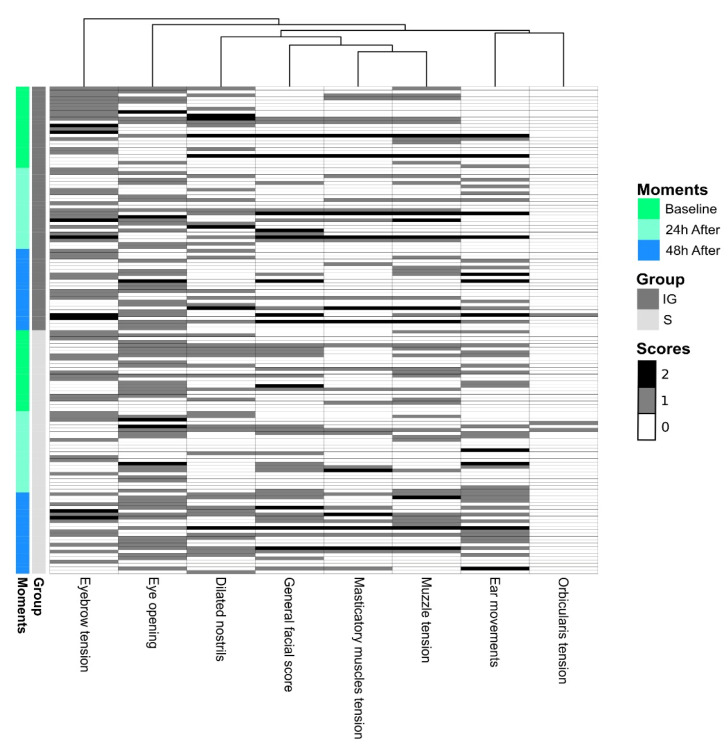
Graphical representation of medians and amplitude of facial expressions and general facial score (GFS) of horses submitted to implantation of polymers. IG, implanted group; S, sham group: skin incision only. The dendrogram (top of the figure) represents the hierarchical grouping, showing the distances between the scoring profiles for each observed variable.

**Figure 4:**
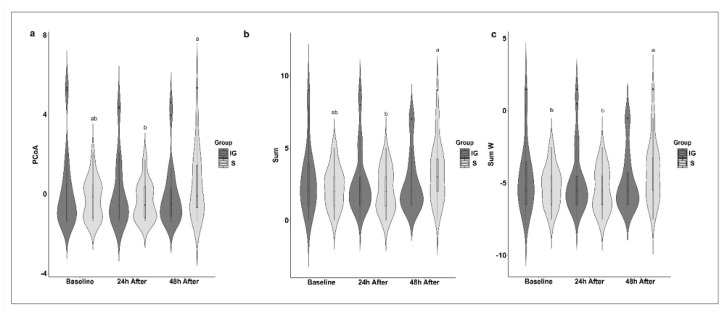
Violin plot of medians and amplitude of PCoA (**a**), SUM (**b**), and SUM.W (**c**) of horses submitted to implantation of polymers. IG, implanted group; S, sham group: skin incision only. Different letters indicate differences over time for the same group (*p* < 0.05).

The FVFs were used to detect nociceptive responses to the implantation of polymers using the mechanical nociceptive threshold (MNT). In the intragroup evaluation, in both experimental groups, the MNT was lower 24 and 48 h after the procedure compared to the baseline (Figure 2 and Table 5). Thermographic evaluation was used to detect alterations in the temperature pattern of the skin surface due to the heat emitted from the inflammatory response induced by implantation. In the S group, the CT was greater 24 and 48 h after the procedure compared to the baseline, while in the IG group, the CT was greater 48 h after the procedure, although there was a significant increase at 24 h (Figure 2 and Table 5). The heatmap shows the clustering of the scores obtained from evaluating each facial expression (FE) and the general facial score (GFS). The black and white gradient analyzes the scores’ contrast, that is, the response intensities observed. The white color indicates a score of 0, and the gray and black colors indicate scores are equivalent to 1 and 2, respectively. The constructed heatmap did not reveal any specific clusters based on the moment and group variables. In addition, the dendrogram (top of the figure) represents the hierarchical grouping, showing the distances between the scoring profiles for each observed variable. In the intergroup evaluation, there were no differences in the facial expressions. However, the CT was higher in the IG group 24 and 48 h after the procedure, while the MNT was lower in the IG group 24 and 48 h after the procedure (Figure 2, Figure 3 and Figure 4 and Table 5). When evaluating each facial feature separately and the GFS, it was not possible to observe intra- or intergroup differences (Figure 3). The SUM, PCoA, and SUM.W were higher 48 h after the procedure for the S group. In the IG group, we did not observe alterations in the statistical analyses performed (Figure 4 and Table 5).

**Figure 5:**
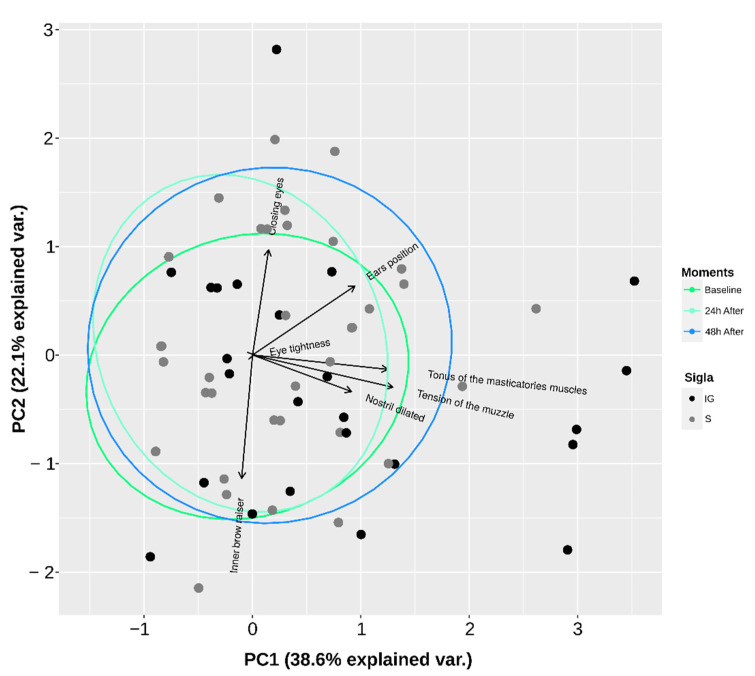
Projections of the loading values in the two dimensions of the principal component analysis performed with the facial expressions evaluated in horses submitted to polymer implantation. IG, implanted group; S, sham group—only skin incision.

The first and second dimensions of the PCoA accumulated more than half of the total variance (52%), and the load values for each facial feature of these two main dimensions are presented in Table 6 and Figure 5.

The variables of the facial expressions showed a very small significant correlation (−0.24 to 0.10) with the CT and MNT. Despite this, other significant correlations were observed (Table 7).

## 4. Discussion

To the best of our knowledge, this is the first study to investigate the ability of facial expression scales to detect pain in horses submitted to polymer implantation. Our findings suggest that the facial expressions were not able to be used to detect pain in the horses, even though all horses implanted with the polymers showed a local acute inflammatory process and skin sensitivity.

The choice to evaluate through video clips was based on the fact that facial expressions are dynamic and often complex and can change rapidly in response to a variety of environmental stimuli and emotional states, with specific movements, such as eyebrow tension and eye tightness, only being accurately distinguished if evaluated in sequence. Thus, the observation of videos may be of higher value for pain assessment [64,70]. Different biases can influence behavioral studies; however, the two prerequisites established for this study were met. The intraobserver reliability demonstrated that our experienced evaluator’s facial evaluation presented substantial to almost perfect repeatability [71]. In addition, in the test retest, there was no difference between the scores of the video clips from the same animal filmed at the same moment, showing that the video clips used as repetitions were equivalent and representative of the state of the animal at the moments evaluated. Therefore, we could understand that the facial features evaluated would not be expressed at a specific time within the total 5 min of recording, representing the state of the horse itself. In general, the test retest is the type of analysis that has not been considered in studies on facial expressions, despite its importance due to the subtlety of facial alterations and the speed with which they can be displayed or not displayed by animals [67].

In a recent study by our research team [65], the same horses were evaluated for six months after the subcutaneous implantation of polylactide-based materials, using established methods to detect inflammation and local sensitivity (MNT and CT). There was an acute and transient low-grade inflammatory process. To characterize if there was painful stimulus here in our opportunistic study, we evaluated the moments 24 and 48 h after the procedure, which were considered the peak of inflammation, based on the previous study [65]. The present study utilized a small number of horses, due to restricted horse availability, since it was an opportunistic study. Since the original study, which aimed to evaluate the biocompatibility and biodegradability of these polymers, was carried out on horses, an animal species that is not discarded at the end of the experiment, few implantations were performed. We decided to perform this study for better use of the research data and animals. In the present study, for both groups, the increase in the CT and reduction in the MNT 24 h and 48 h after implantation indicated an inflammatory process, which was interpreted as originating from the surgical procedure itself. The higher temperatures and lower MNTs observed in the IG group suggested a higher degree of an inflammatory response, which was expected because the implant material is exogenous to the organism, despite having shown biocompatibility [65,72,73]. Another possible explanation for this could be that implanted polyester biomaterials, such as PLA and PCL, would progressively release some acidic degradation products [74]. It could reduce the local pH, which could induce chemical leads via the release of pro-inflammatory mediators, evoking pain and a more evident inflammatory process [75].

Despite the inflammatory process and increase in local sensitivity found in our study, the strategies customarily used to analyze facial expressions (SUM and individual alterations in facial features) showed few changes after the procedure. Here, it is important to highlight that the recording of the facial expressions was performed with the horses standing still and not when we were inducing stimulus with the FVF, so it is possible, even likely, that there was no spontaneous/ongoing pain during the filming. It was expected that the increase in SUM, SUM.W, and PCoA 48 h after the procedure, as shown for the S group, would also be observed in the IG group. However, this did not occur. In the group with a more evident inflammatory process (IG), the facial expressions did not alter after the procedure. This could partially be explained by confounding factors, which may hinder the estimation of pain in horses [7].

It is important to consider that some facial features of pain can be affected by other cognitive states, so caution is needed when using them in other situations, for example, stressful situations, since simple management procedures could induce similar facial expressions [55,64]. For example, dilated nostrils can be perceived as pain in the postoperative period [45] and in induced orthopedic pain [57]. On the other hand, dilated nostrils were also observed after intense physical exercise [51] or stressful management conditions [55]. This can be explained by the fact that the respiratory rate increases during stress, pain, and exercise [55]. Another example is partially or entirely closed eyes that have been associated with post-castration pain [45], physical tiredness [51], exhaustion due to anesthetic and surgical procedure [47], relaxation and positive emotions [50], sleep or rest [61], or sedation [56]. Finally, eyebrow tension, which can occur as a response to painful stimulus [47], can also be expressed when the horse is curious, attentive, afraid, or surprised [61]. Based on these interrelationships mentioned above, if facial expressions are used individually as an indicator of pain, it is possible they would have high sensitivity, as they can be expressed in painful moments, but low specificity, because they can be displayed in pain-free moments due to other sensations that would trigger them. Thus, facial features cannot be interpreted singularly [62,63], eliminating false-positive diagnoses and the overestimation of pain.

Furthermore, there are some behaviors that can be influenced by the time of the day, such as increased walking, standing still, looking out the window during the day, and increased pelvic limb resting during the night [7], and the same can happen with facial expressions. For example, partially closed eyes can happen at night, because the horses are sleeping or resting [61], or, as we saw in this study, in the early morning, and can be confused with pain.

In this sense, we tested different strategies for evaluating facial expressions to deeply understand the findings obtained. Some of these strategies have not previously been applied in studies that used the facial evaluation of horses, for example, the extraction of the principal component analysis (PCoA) scores, the application of specific weightings to each face characteristic (SUM.W), and a general face score (GFS). In the first dimension of the principal component analysis, the masticatory muscles tension showed the highest loading value, highlighting its greater importance in the data set, followed by muzzle tension. In the same way, studies that have investigated pain in horses using the face have indicated an increase in these features at painful moments [45,46,47,48,49]. Thus, among the facial features evaluated in our study, the masticatory muscles and muzzle tension may be more relevant for pain evaluation in horses.

The differential of the PCoAs or SUM.W is that they consider a set of facial features that, as far as we know, has never been tested before, and we could denote it as a facial configuration. As previously explained, eyes partially or fully closed may indicate pain but may also indicate other sensations. However, when this specific characteristic is being exhibited concurrently with others that also signal pain, such as tension in the muzzle, eyebrows, and masticatory muscles, we could say that there is a display of pain in the facial configuration that comprises an integrated investigation of facial action units, which is an innovation presented in our study.

Our proposal for pain facial configuration is also based on the fact that, in general, animals in pain remain more immobile but tense [6,11,76], as just immobility would apply to a facial configuration of sleepiness or relaxation when the animal has closed eyes, muzzle, or lips and relaxed masticatory muscles [50,54,55]. On the other hand, a study showed that horses were more restless during pain induction [47]. Besides the biological reasons for using the scores obtained from PCoA or SUM.W, in the interpretation of facial expressions, there are also mathematical gains, such as the transformation of discrete variables (e.g., scores of 0, 1, and 2 or scores referring to the sum of a scale) into continuous variables. With this, it would be possible to find less variability in the data set and the application of more robust parametric statistical analyses than non-parametric data.

In the current study, it was not possible to evidence the presence of a pain face configuration using any strategy, which suggests that the polymer implantation may not have induced subtle pain or low-grade inflammatory pain in our horses. This finding can be partially explained by the complexity of pain evaluation in horses, which involves somatic, cognitive, and emotional components [2,3], and could be more complicated in subtle or mild pain [4,7]. In humans, it has been reported that facial expression in response to painful stimuli is less prominent in weak stimuli compared to strong stimuli, so it was difficult for the observer to detect the pain grade when the subjects felt mild pain [77].

A possible explanation for the lack of efficiency in pain behavior detection, by all five facial expression evaluation strategies adopted herein, is that spontaneous and subtle pain may not be ongoing, unlike induced pain or pain that originates postoperatively in more invasive procedures, which are used as a pain model by other studies with facial expressions in horses [4,7,45,46,47,48,52,57,64]. It is assumed that when the horse remains with its neck still, as in our filming, it does not feel pain or, perhaps, only slight discomfort. The animal could more easily exhibit pain when there is movement or manipulation of the inflamed area, as during the MNT test. Corroborating our reasoning, some authors state that the reaction triggered by palpation does not necessarily correlate with the level of pain experienced when the area is left untouched and, therefore, should be interpreted with caution [6]. Studies comparing the effects of hot iron branding and microchip transponder injection in adult horses [78] and foals [79] detected behavioral alterations associated with stress/pain, since, in both studies, the animals were observed while the procedures were performed.

In the same way, facial expressions at rest for horses with induced orthopedic pain can vary between horses and are of less value, since lame horses can modify their pain at rest by avoiding situations that may increase pain intensity, e.g., by decreasing the load on the painful limb. Furthermore, several other factors can lead to pain not being detected, such as the animal’s personality, which can influence the way it expresses pain, the presence of possible threats in the environment, which can lead the animal to suppress pain, the animal’s age, the type of pain, other emotional states, etc. In addition, the small number of horses used herein could have led to individuals’ variations in facial expressions, since pain is an experience related to personality, and it is not expected that all horses show the same expressions [57]. Moreover, it is important to understand that all behaviors and facial expressions are part of the non-verbal communication system of healthy horses, and it is their combination and frequency that can indicate if pain is present [4]. Thus, the gap in the results for pain detection in our study demonstrates the complexity of pain evaluation in some cases and a clear need for further studies focusing on pain of this nature.

Some limitations and implications of our work need to be recognized. In future studies, the proposed mathematical approaches could be tested on horses submitted to different pain intensities, in a dose vs. response type of study, with the video clips being watched by more than one evaluator to estimate the reproducibility (interobserver reliability). Comparisons of video and loco evaluations could also be performed. The small number of animals or the 12 lateral surfaces of the neck used herein could be a limitation for the principal component analysis. However, the PCA was performed following the indications of at least five observations per variable [80], even though studies with a more significant number of animals should be accomplished.

Regarding practical implications, our findings suggest that the application of facial expressions as a generalized tool to recognize nociception or subtle pain in horses must be carried out with caution. Mild or subtle pain should be further studied because, although to a lesser extent, it can represent a source of animal suffering that may be overlooked or underestimated, since they may feel pain only when the lesion is being stimulated. Nonetheless, pain in relation to palpation is relevant data; for example, if palpation or manipulation is painful, the analgesic treatment protocol may be changed, or local analgesics may be implemented [6]. Finally, more and more, this care is becoming relevant in the current global scenario, due to growing concern by society for the quality of life of non-human animals.

## 5. Conclusions

The findings of this research show that none of the five methodological strategies used to evaluate facial configurations could detect nociception or subtle pain in horses. It is challenging to detect such pain, since the resulting pain behavior is also subtle, sparsely appearing, and varying, so it could be more easily exhibited by the animal when there is movement or manipulation in the inflamed area. Therefore, the relationship between facial configuration and nociception or subtle pain needs to be better studied in future studies.

## Figures and Tables

**Table 2:** Behavioral categories considered aversive during the assessment of the mechanical nociceptive threshold.

Behavioral Categories	Descriptions
Tail movement	Moves the tail to the left or right above or below the groin or up and down at least 45 degrees quickly.
Ear movement	Both ears are turned laterally and dorsally/caudally. The inner ear opening faces outward.
Head movement	Throws the head up or down, with or without moving the neck in the same direction simultaneously. Turns the head to the right or to the left, leaving the whites of the eye visible. Turns the head toward the stimulus.
Kick or step sideways	Moves with two legs, forward, backward, or sideways, resulting in a new position, or raises the pelvic member and vigorously moves it backwards or sideways.

**Table 3:** Intraobserver reliability (repeatability) estimated by the quadratic weighted kappa coefficient and its 95% confidence interval calculated for the 7 facial features.

		95% Confidence Interval	
Facial Features	Weighted Kappa	Minimum	Maximum
Ear movements	0.78	0.39	1.00
Eyebrow tension	0.71	0.47	0.96
Orbicularis tension	1.00	1.00	1.00
Dilated nostrils	1.00	1.00	1.00
Eye opening	1.00	1.00	1.00
Muzzle tension	0.86	0.66	1.00
Masticatory muscles tension	1.00	1.00	1.00

**Table 4:** Median and amplitude (minimum–maximum) of the 7 facial features and the general facial score (GFS) in each of the two video clips of the same animal filmed at the same time to evaluate the retest.

	Video Clip 1		Video Clip 2	
	Median	Range	Median	Range
Ear movements	0	0–2	0	0–2
Eyebrow tension	1	0–2	0	0–2
Orbicularis tension	0	0–1	0	0–1
Dilated nostrils	1	0–2	1	0–2
Eye opening	0	0–2	0	0–2
Muzzle tension	0	0–2	0	0–2
Masticatory muscles tension	0	0–2	0	0–2
GFS	0	0–2	0	0–2

**Table 5:** Median and amplitude (minimum–maximum) of the nonparametric variables (**A**) and mean and standard deviation of the variables assumed to be parametric (**B**) before and 24 and 48 h after the procedure. Different capital letters indicate a statistical difference between the groups. Different lowercase letters indicate an intragroup statistical difference over time. *p* < 0.05. Being that a > b > c and A > B.

A							
		Before		24 h After		48 h After	
Groups	Variables	Median	Amplitude	Median	Amplitude	Median	Amplitude
S	Ear movements	0.00	0–1	0.00	0–2	1.00	0–2
	Eyebrow tension	0.00	0–1	0.00	0–1	0.00	0–2
	Orbicularis tension	0.00	0–0	0.00	0–1	0.00	0–0
	Dilated nostrils	0.00	0–1	0.00	0–1	0.50	0–2
	Eye opening	1.00	0–1	0.50	0–2	1.00	0–1
	Muzzle tension	0.00	0–1	0.00	0–1	0.00	0–2
	Masticatory muscles tension	0.00	0–1	0.00	0–2	0.00	0–2
	SUM	2.00 ab	0–5	2.00 b	0–5	3.00 a	0–9
	PCoA	−0.15 ab	−1.35–2.08	−0.56 b	−1.35–1.73	−0.08 a	−1.35–5.34
	SUM.W	−5.54 b	−7.54–−2.54	−5.54 b	−7.54–−2.54	−4.54 a	−7.54–1.46
	GFS	0.00	0–2	0	0–1	0.5	0–2
	MNT	446.68 a	446.68–446.68	203.87 bA	11.75–446.68	281.84 bA	15.14–446.68
IG	Ear movements	0.00	0–2	0.00	0–2	0.00	0–2
	Eyebrow tension	1.00	0–2	1.00	0–2	0.00	0–2
	Orbicularis tension	0.00	0–0	0.00	0–0	0.00	0–1
	Dilated nostrils	0.00	0–2	0.00	0–2	0.00	0–2
	Eye opening	0.00	0–2	0.50	0–2	1.00	0–2
	Muzzle tension	0.00	0–2	0.00	0–2	0.00	0–2
	Masticatory muscles tension	0.00	0–2	0.00	0–2	0.00	0–2
	SUM	2.00	0–9	2.00	0–9	2.00	1–7
	PCoA	−0.59	−1.42–5.34	−0.57	−1.35–4.39	−0.52	−1.42–4.60
	SUM.W	−5.54	−7.54–1.46	−5.54	−7.54–1.46	−5.54	−6.54–−0.54
	GFS	0.00	0–2	0.00	0–2	0.00	0–2
	MNT	446.68 a	446.68–446.68	75.86 bB	28.84–281.84	77.37 bB	11.75–281.84
**B**							
		**Before**		**24 h After**		**48 h After**	
		**Mean**	**SD**	**Mean**	**SD**	**Mean**	**SD**
S	CT	32.92 b	1.10	34.38 aB	0.69	34.32 aB	0.87
IG	CT	33.46 b	0.92	35.29 aA	0.48	35.75 aA	0.58

**Table 6:** Loading values of each facial feature in the first two dimensions extracted from the principal component analysis.

	Dimension 1	Dimension 2
Ear movements	0.59	0.39
Eyebrow tension	−0.06	−0.76
Orbicularis tension	0.06	0.03
Dilated nostrils	0.10	0.75
Eye opening	0.87	−0.07
Muzzle tension	0.67	−0.22
Masticatory muscles tension	0.85	−0.17
Eigenvalue	2.29	1.36
Variance (%)	32.83	19.52

**Table 7:** Spearman rank correlation matrix between the mechanical nociceptive threshold, cutaneous temperature, and strategies for evaluating facial expressions (the 7 individual features, SUM, SUM.W, PCoA, GFS).

	MNT	CT	Ear Movements	Eyebrow Tension	Orbicularis Tension	Dilated Nostrils	Eye Opening	Muzzle Tension	Masticatory Muscles Tension	GFS	SUM	PC	SUM.W
MNT													
CT	−0.55 *												
Ear movements	−0.13	0.02											
Eyebrow tension	0.05	−0.08	−0.22 *										
Orbicularis tension	−0.13	0.06	0.03	0.00									
Dilated nostrils	−0.02	−0.10	0.13	0.07	−0.01								
Eye opening	−0.20 *	0.03	0.18 *	−0.30*	0.03	0.02							
Muzzle tension	0.03	−0.06	0.27 *	0.07	0.08	0.38 *	0.02						
Masticatory muscles tension	−0.16	−0.06	0.28 *	−0.06	0.03	0.40 *	0.02	0.62 *					
GFS	−0.24 *	−0.02	0.45 *	−0.08	0.12	0.34 *	0.27 *	0.54 *	0.62 *				
SUM	−0.13	−0.07	0.51 *	0.22*	0.09	0.61 *	0.36 *	0.69 *	0.62 *	0.63 *			
PCoA	−0.11	−0.05	0.52 *	−0.14	0.09	0.62 *	0.26 *	0.79 *	0.71 *	0.65 *	0.87 *		
SUM.W	−0.13	−0.07	0.51 *	0.22*	0.09	0.61 *	0.36 *	0.69 *	0.62 *	0.63 *	1.00 *	0.87 *	

The numbers in bold represent correlation > 0.50 or < −0.50, and * indicates *p* < 0.05.

## Data Availability

The raw/processed data required to reproduce these findings are available from the corresponding author upon reasonable request.
